# Sexual maturation in hens is not associated with increases in serum leptin and the expression of leptin receptor mRNA in hypothalamus

**DOI:** 10.1186/2049-1891-4-24

**Published:** 2013-06-26

**Authors:** Yingdong Ni, Jinfang Lv, Shaoqing Wang, Ruqian Zhao

**Affiliations:** 1 Key Laboratory of Animal Physiology & Biochemistry, Nanjing Agricultural University, Nanjing 210095, P. R. China; 2 Animal Science College, Anhui Science and Technology University, Fengyang 233100, P. R. China

**Keywords:** Gonadatrophin Releasing hormone (GnRH), Hens, Hypothalamus, Leptin, Sexual maturation

## Abstract

**Background:**

In mammals, leptin is an attractive candidate for mediating the metabolic signal and the reproductive function via the specific receptor in hypothalamus. However, till now, the role of leptin on reproduction in birds is less well established. This experiment was conducted to elucidate the role of leptin on the onset of reproduction in bird, as a first step, to detect the changes of peripheral leptin and leptin receptor mRNA expression in hypothalamus between mature and immature hens at the same age. 120 ISA brown pullets at D60 were allocated randomly into two groups, long light (LL) group being raised under artificial light regimes with incrementally increased light phase (from 8 L:16D to 14 L:12D) and short light (SL) group raised on consistent light (8 L:16D) for 12 wk.

**Results:**

The results showed that pullets in LL group reached sexual maturation 15 d earlier than those in SL group. Serum E_2_ showed a significant increase with age, but no difference was observed between two groups. Serum leptin concentration decreased significantly from D112 to D136 in LL, and was markedly higher in LL group than that in SL at D112, while there was no significant difference between two groups at D136. Leptin receptor and GnRH-I mRNA expression in hypothalamus were significantly increased with age, yet there was no significant difference between SL and LL chickens at the same age. The expression of FSH-β and LH-β mRNA in pituitary was increased with age but did not show significant difference between LL and SL group. GnRH-I mRNA expression was very rich in pineal gland, and decreased from D112 to D136 in LL but not in SL group, and there was no difference between two groups at the same age.

**Conclusions:**

These results indicate that the earlier sexual maturation in hens induced by long-light regime is not accompanied with an increase in serum leptin or leptin receptor gene expression in hypothalamus, or genes expression in HPG axis.

## Background

In the 1970s, the frequently observed relationship between weight and age at menarche was put together by Frisch as so called ‘critical weight hypothesis’, which states that a certain body fat depot seems to be required for the process of initiating normal reproductive function [1]. Leptin, a hormone derived from adipocytes, which is encoded by the ob gene, is an attractive candidate for mediating this effect [2]. Leptin circulates in concentrations proportional to adiposity and serves as a negative feedback signal to the hypothalamus through suppression of appetite and stimulation of energy expenditure [3-5]. In addition, leptin may act as a metabolic signal involved in the control of the onset of sexual maturation and reproduction via leptin receptor in hypothalamus to activate GnRH secretion. Chronic leptin injection can restore fertility in the leptin deficient ob/ob mice [6,7]. Nevertheless, there has been considerable controversy as to whether leptin serves a permissive or a triggering role in this regard. Only a single study reports that exogenous leptin injection can promote the sexual maturation in domestic hen [8]. However, until now, the role of leptin on reproduction in birds is less well established.

As an important blood-borne metabolic signal timing the pubertal increase in gonadatrophin releasing hormone (GnRH) secretion, the circulating substance must be quantitatively different for the sexually mature and sexually immature individual [9]. In fact, serum leptin concentrations increased during puberty in the mouse [10], heifer [11] and pig [12]. In pig, during pubertal development, serum leptin levels and hypothalamic leptin receptor mRNA expression increased with age [12]. Up to today, the relevant studies in bird are really scarce. Whether in bird leptin activates the reproductive axis or serves as a permissive factor, whose presence is required as suggested the role in mammalian species, it is still a mystery. In order to answer this question, as the first step, the measurements of circulating leptin concentration and central leptin receptor mRNA expression, as well as GnRH-I gene expression in the hypothalamus and pineal gland between mature and immature birds at the same age was detected in this study.

## Methods

### Animals and experimental design

One hundred and twenty female ISA laying hens (60-day-old, D60) were allocated randomly into 2 groups (3 replicates per group), which were raised under artificial light regimes with incremental increased long light (LL, from 8 L:16D to 14 L:12D over 12 wk) or consistent short light (SL, 8 L:16D) from D60. The body weight and serum E_2_ concentration had no difference between two groups on D60 (data not shown). Standard commercial diets and water were available *ad libitum*. The laying rate of the flock reached 5% at D136 in LL and at D151 in SL group, respectively, and showing 15 d difference of sexual maturation between two groups caused by different light regimes. Blood were collected on D112 and D136 (n = 20), respectively. The sera were stored at −20°C until hormone assays were conducted. The tissues of hypothalamus and pineal gland were collected (n = 10) at the same time of day, and then was snap frozen in liquid nitrogen for isolation total RNA.

Animal care and use above was approved by the Institutional Animal Care and Use Committee of Nanjing Agricultural University.

### Q-RT-PCR assay for GnRH-I gene expression in pineal gland

Total RNA was extracted from pineal gland tissues using the Trizol RNA easy Kit (Qiagen) procedure and quantitated at 260 nm, with acceptable 260/280 ratios of >1.8. MMLV reverse transcriptase (RT) (Promega, USA) was used to reverse transcribe 2 μg of total RNA, using an oligoT_15_ primer in a total reaction volume of 25 μL to obtain cDNA. After the RT reaction, aliquots of 2 μL from pineal gland tRNA for GnRH-I was used as cDNA template for polymerase chain reaction (PCR) amplification, in duplicate. The thermal cycling parameters used for GnRH-I were: 30 cycles, 94°C for 30 s, 55°C for 30 s, and 72°C for 30 s. 25 μL PCR reactions included the chicken GnRH-I specific primer pair; forward primer 5′-GCTTGGCTCAACACTGGTCT-3′; backward primer 5′-CTGGCTTCTCCTTC-GATCAG-3′, that produce a 202-base pair (bp) amplification product and the product was sequenced by TaKaRa Biotechnology (TaKaRa, Dalian, China). As an internal control for the integrity of the mRNA in each sample, an additional primer pair specific for chicken β-actin that produce a product (268 bp) was amplified in the same run with PCR reactions for chicken GnRH-I. The pooled samples made by mixing equal quantity of total RNA from all samples were used for optimizing the PCR condition and normalizing the intra-assay variations. All samples were repeated at least 3 times. An aliquot 15 μL of PCR products was analyzed by electrophoresis on 2% agarose gels stained with ethidium bromide. The net intensities of individual bands were measured using Kodak Digital Science 1D software (Eastman Kodak Company Rochester, NY, USA).

### Real-time RT-PCR for genes expression in hypothalamus and pituitary

Real-time PCR was performed in Mx3000P (Stratagene, USA). Mock RT and No Template Controls (NTC) were set to monitor the possible contamination of genomic DNA both at RT and PCR. The pooled sample made by mixing equal quantity of total RT products (cDNA) from all samples was used for optimizing the PCR condition and tailoring the standard curves for each target gene, and melting curves were performed to insure a single specific PCR product for each gene. Two microliter of 16-fold dilution of RT product was used for PCR in a final volume of 25 μL containing 12.5 μL SYBR Green Real-time PCR Master Mix (TOYOBO Ltd., Japan) and 0.4-1.0 μmol/L of each forward and reverse primers for cGnRH-I, leptin R, LH and FSH genes (the information of primers was shown in Table 1). Chicken β-actin mRNA was used as a reference gene for normalization purpose. The following PCR protocols were initial denaturation (1 min at 95°C), then a three-step amplification program (20 sec at 95, 20–30 sec at 60–64, 20 sec at 72°C) was repeated 45 times. The PCR products for each gene were sent to Haojia Biotech, Ltd., for sequencing to verify the specificity. The reported sequences exactly matched those published in GenBank. The method of 2^-ΔΔCt^ was used to analyze the real-time RT-PCR data. All samples were included in the same run of RT-PCR and repeated in triplicates.

**Table 1:** Nucleotide sequences of specific primers

**Target genes**	**GenBank accession No.**	**Products**	
		**Length (bp)**	**Primer sequences**
cGnRH-I	NM_001080877.1	202	F:5′-GCTTGGCTCAACACTGGTCT-3′
			R:5′-CTGGCTTCTCCTTCGATCAG-3′
Leptin R	AF169827	97	F:5′-GCTTGCTCAGGTAGCTCCTG-3′
			R:5′-TGCGGCACGTATGGCACGAT -3′
LH (β)	HQ872606.1	223	F:5′-GTATGGCTGTGACCACCACGG-3′
			R:5′-CTGCACGGTGCAGTCGGAG-3′
FSH (β)	NM_204257.1	142	F:5′-CGTGGTGCTCAGGATAC-3′
			R:5′-AAAAGATTCAGGATGGT-3′
β-actin	NM_205518.1	299	F:5′-TGCGTGACATCAAGGAGAAG-3′
			R:5′-TGCCAGGGTACATTGTGGTA-3′

### Radioimmunoassay for serum hormone levels

The serum E_2_, T_3_ and T_4_ were measured with radioimmunoassay (RIA) using commercial kits purchased from Beifang Institute of Biological Products (Beijing China). Serum leptin was detected by a kit bought from Shanghai Institute of Biological Products (Shanghai, China) as previously described by Hu *et* al. [13]. The ranges of assay sensitivity for E_2_ were between 5 and 4,000 pg/mL. The internal and intra-assay coefficient of variation were 10% and 15%, respectively. The ranges of assay sensitivity were 0 to 8.0 ng/mL for T_3_ and 10 to 200 ng/mL for T_4_. The ranges of assay sensitivity for leptin were between 0.5 and 24 ng/mL. The internal and intra-assay coefficient of variation were 5% and 10%, respectively.

### Statistic analysis

All statistical analyses were performed with SPSS 11.0 for Windows (SPSS Inc., Chicago, IL). The results were expressed as mean ± S.E.M and differences were considered significant when *P* < 0.05 tested by ANOVA with SPSS 11.0 for windows. Numbers used for statistics are noted in the table and figures. 

## Results

### Comparison of body weights, ovary weights, the ratio of ovary weight to body weight, E_2_ and leptin in serum between LL and SL hens

As shown in Tables 2 and 3, there was no significant difference of body weight, ovary weight and the ratio of ovary weight to body weight at D112 between LL and SL hens (*P>0.05, n = 20*), however, at D136, these indexes were statistically higher in LL group than that in SL (*P<0.05, n = 20*). Serum leptin concentration decreased significantly from D112 to D136 in LL hens (*P<0.05, n = 20*), which was significantly higher in LL hens than that in SL at D112 but not at D136. For hens in SL group, the level of serum leptin was not shown significant difference with age (*P>0.05, n = 20*). Except a significant decrease of serum T_3_ concentration in LL group at d136 compared to SL group, there was no significant changes of serum E_2_ or T_4_ between LL and SL hens at each corresponding age (*P>0.05, n = 20*). Tables 2 and 3.

**Table 2:** Body weight, ovary weights and the ratios of ovary weight to body weight of LL and SL hens

	**D112**		**D136**	
**Parameters**	**LL**	**SL**	**LL**	**SL**
Body weight (Kg)	1.31 ± 0.03^a^	1.29 ± 0.04^a^	1.58 ± 0.04^b^	1.43 ± 0.03^c^
Ovary weight (g)	0.47 ± 0.04^a^	0.50 ± 0.03^a^	16.42 ± 3.55^b^	0.76 ± 0.16^a^
Ovary weight/body weight (g/kg)	0.61 ± 0.05^a^	0.65 ± 0.05^a^	26.34 ± 5.57^b^	1.11 ± 0.26^a^

Values are means ± S.E.M. *LL* long-light group, *SL* short-light group.

Values in the same experiment bearing different superscripts (a, b, c) are significantly different (*P* < 0.05, n = 20).

**Table 3:** **E**_**2**_**, leptin, T**_**3**_**, T**_**4 **_**hormone levels and the ratio of T**_**3**_**/T**_**4 **_**in serum of LL and SL hens**

	**D112**		**D136**	
**Parameters**	**LL**	**SL**	**LL**	**SL**
E_2_ (ng/ml)	248.08 ± 19.76^a^	281.76 ± 18.53^a^	334.20 ± 12.25^b^	357.35 ± 9.04^b^
Leptin (ng/ml)	1.51 ± 0.07^a^	1.27 ± 0.08^b^	1.03 ± 0.05^c^	1.14 ± 0.07^bc^
T_3_ (ng/mL)	4.04 ± 0.30^ab^	4.52 ± 0.41^a^	1.62 ± 0.12^c^	3.42 ± 0.42^b^
T_4_ (ng/mL)	37.61 ± 1.15^a^	34.15 ± 1.95^ab^	30.96 ± 1.18^b^	36.14 ± 1.26^ab^

Values are means ± S.E.M. *LL* long-light group, *SL* short-light group.

Values in the same experiment bearing different superscripts (a, b, c) are significantly different (*P* < 0.05, n = 10).

### Gene Expressions in pineal gland, hypothalamus and pituitary

**Figure 1:**
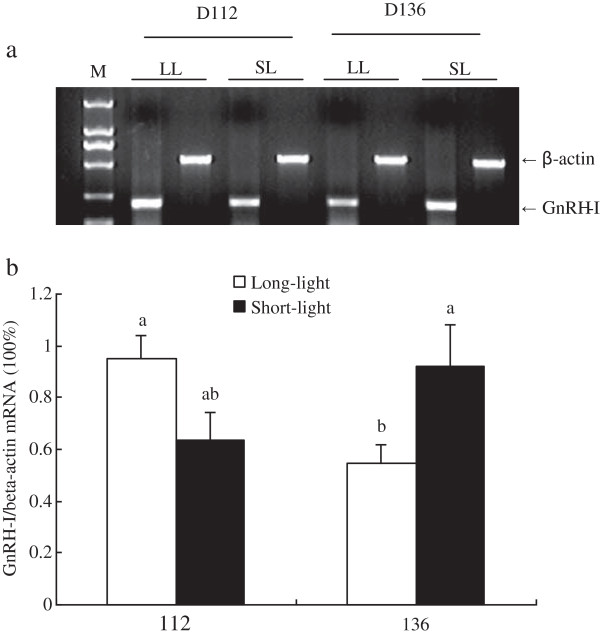
**GnRH-I mRNA expression in pineal gland between LL and SL chickens at D112 and D136.** (**a**) Representative electrophoresis photo of RT-PCR products for GnRH-I and β-actin mRNA, respectively. M: DNA molecular weight marker DL2000. LL and SL lanes stand for long-light and short-light, respectively. (**b**) Results of statistical analysis for GnRH-I mRNA level expressed as arbitrary units relative to β-actin mRNA. The results were expressed as mean ± S.E.M and differences were considered significant when *P* < 0.05 tested by ANOVA with SPSS 11.0 for windows. Mean values with (**a**, **b**) differ significantly between LL and SL groups (*P* < 0.05, n = 10).

**Figure 2:**
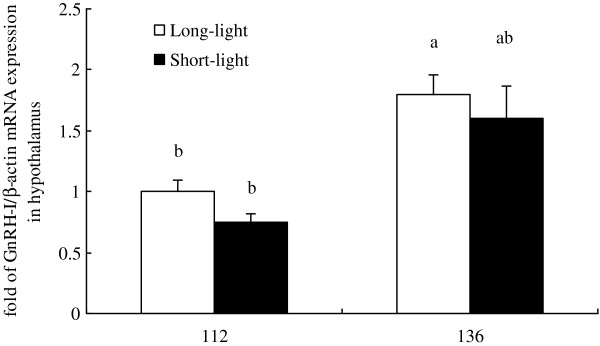
**GnRH-I mRNA expression in hypothalamus between LL and SL chickens at D112 and D136.** Results of statistical analysis for GnRH-I mRNA level expressed as arbitrary units relative to β-actin mRNA. The results were expressed as mean ± S.E.M. and differences were considered significant when *P* < 0.05 tested by ANOVA with SPSS 11.0 for windows. Mean values with (**a**, **b**) differ significantly between LL and SL groups (*P* < 0.05, n = 10).

**Figure 3:**
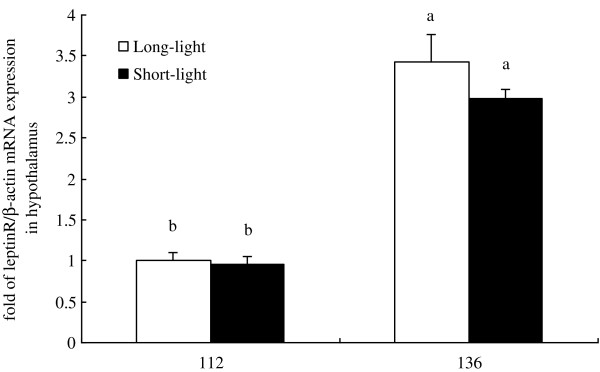
**Leptin receptor mRNA expression in hypothalamus between LL and SL chickens at D112 and D136.** Results of statistical analysis for leptin receptor mRNA level expressed as arbitrary units relative to β-actin mRNA. The results were expressed as mean ± S.E.M. and differences were considered significant when *P* < 0.05 tested by ANOVA with SPSS 11.0 for windows. Mean values with (**a**, **b**) differ significantly between LL and SL groups (*P* < 0.05, n = 10).

**Figure 4:**
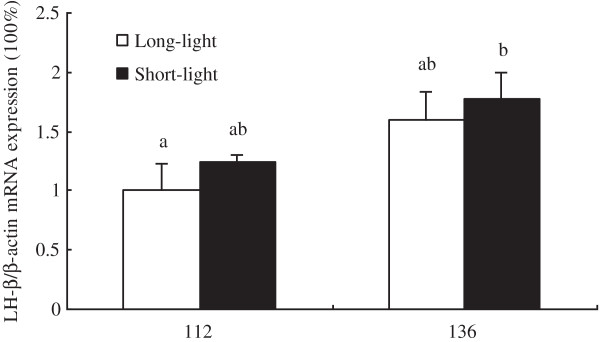
**LH-β mRNA expression in pituitary between LL and SL chickens at D112 and D136.** Results of statistical analysis for LH-β mRNA level expressed as arbitrary units relative to β-actin mRNA. The results were expressed as mean ± S.E.M. and differences were considered significant when *P* < 0.05 tested by ANOVA with SPSS 11.0 for windows. Mean values with (**a**, **b**) differ significantly between LL and SL groups (*P* < 0.05, n = 10).

**Figure 5:**
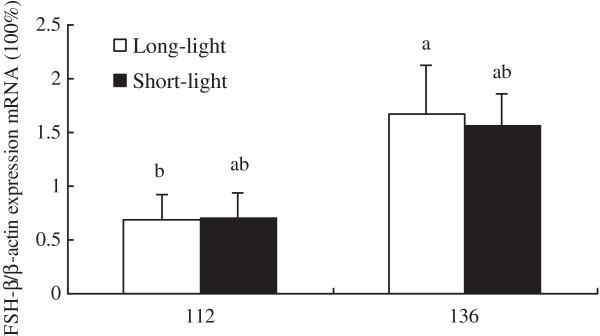
**FSH-β mRNA expression in pituitary between LL and SL chickens at D112 and D136.** Results of statistical analysis for FSH-β mRNA level expressed as arbitrary units relative to β-actin mRNA. The results were expressed as mean ± S.E.M. and differences were considered significant when *P* < 0.05 tested by ANOVA with SPSS 11.0 for windows. Mean values with (**a**, **b**) differ significantly between LL and SL groups (*P* < 0.05, n = 10).

As shown in Figure 1–5, at the age of D136, the level of GnRH-I mRNA expression in pineal gland of LL birds was significantly lower than that at D112, and was also markedly lower than un-mature SL chickens at D136 (*P<0.05, n = 10*). Within SL group, there was no difference of GnRH-I mRNA expression in pineal gland between D112 and D136 (*P>0.05, n = 10*) (Figure 1). Hypothalamic GnRH-I mRNA expression significantly increased with age in both groups (*P<0.05, n = 10*), however, there was no difference between LL and SL at the corresponding age (*P>0.05, n = 10*) (Figure 2). Figures 3, 4, 5 showed that leptin receptor mRNA expression in the hypothalamus as well as LH-β and FSH-β mRNA expression in pituitary showed the same pattern as GnRH-I gene in hypothalamus, which increased with age but showed no difference between two groups at the same age (*P>0.05, n = 10*). Figures 1, 2, 3, 4, 5.

## Discussion

The discovery of leptin has improved our understanding of the relationship between energy homeostasis and pubertal initiation. Last decades, particular attention was paid to the effect of leptin on reproduction, especially on the gonadal dysfunction as infertility, a characteristic feature of both *ob/ob* (leptin deficient) and *db/db* (leptin receptor mutant) mice [2,6,7]. Nevertheless, there has been considerable controversy as to whether leptin plays a precise role on reproduction. Recently, Paczoska reported that exogenous injection of recombinant chicken leptin significantly advanced the onset of puberty, and that this advancement was associated with attenuation of ovarian apoptosis [8], which indicates that, as in mammalian animals, leptin is also involved in regulating sexual maturation of the domestic hen.

As an important blood-borne metabolic signal timing the pubertal GnRH secretion, it must be quantitatively different between the sexually immature and the sexually mature individuals, and must increase/decrease during growth [9]. In fact, serum leptin concentrations increased during puberty in the mouse [10], heifer [11] and pig [12]. In this study, there was no significant difference of serum leptin concentration between sexually mature and sexually immature hens. Our results also showed that serum leptin concentrations decreased significantly when approaching to the initiation of sexual maturation in LL hens, which was consistent with the results derived from female mice [14]. With a good agreement with our results, Bronson reported that there is no support for the hypothesis that an increase in body fat and thus an increase in circulating leptin trigger puberty in female mice [14]. Also, thyroid hormones are essential for photoinduced egg production [15] as well as being important for growth and development in vertebrates [16]. In the present study, photo-induced earlier egg production in hens was not accompanied the changes of thyroxine (T_4_).

It is well known that the effects of leptin on regulating reproduction is mediated by its receptor, which was distributed extensively in reproductive axis in most animal species, including avian animals [17]. If the onset of puberty is preceded by an increase in the sensitivity of the hypothalamus-pituitary-gonadal axis to leptin, then leptin could still act as a pubertal trigger in spite of the absence of an obvious prepubertal rise in circulating leptin concentrations. To address this possibility, real-time RT-PCR assay was employed in our current study to investigate whether there are changes of the leptin long form receptor mRNA expression in hypothalamus between sexually mature and immature hens. Our results showed that there was no significant changes of leptin long form receptor mRNA expression in hypothalamus between sexually mature and immature hens, which was consistent with the results from the female rat [18]. However, in pig, during pubertal development, hypothalamic leptin receptor mRNA expression increased with age [12]. The above divergent results may be related to the different animal species which underlying pubertal processes are not identical.

Sexual development and mature reproductive function are controlled by a decapeptide, gonadatrophin hormone releasing hormone (GnRH) secreted from the basal forebrain to stimulate the luteinizing hormone and follicle-stimulating hormone secretion from pituitary. It is well known that GnRH-I plays a critical role in the reproduction of birds [19]. Two types of GnRH, GnRH-I and GnRH -II are secreted from avian hypothalamus. In addition, pineal gland may be another important source for GnRH. Our previous results showed that GnRH-I mRNA expression was rich in pineal gland of female ducks and the nucleotide sequence of 202 base pair length including encoding region shared 99% identity to that of hens [20]. There was a progressive increase of GnRH-I gene expression in hypothalamus but not in pineal gland during sexual maturation in female Shaoxing laying ducks [20,21]. In this study, the results showed that there was no significant difference of GnRH-I mRNA abundance in the hypothalamus between mature and immature hen, which was consistent with LH and FSH genes expression in pituitary as well as the level of serum estrogen between LL and SL hens at D136. In male mouse and rat, synthetic capacity is present well before puberty in that GnRH mRNA expression reaches the adult levels [22]. Moreover, any changes in GnRH content are more likely to reflect the post translational mechanism, rather than changes in the primary transcription rate [22]. However, the existence of mRNA and changes of GnRH-I gene transcription in pineal gland awaits further study.

Although this finding may not support leptin as a hormonal factor triggering sexual maturation in birds, we also observed a significant rise in leptin on D112 in LL hens, which was consistent with our previous studies on ducks [23]. Therefore, it is still possible that leptin may play a role in promoting sexual maturation in avian species already from early postnatal period. As in dairy cattle [24], a sudden increase in plasma leptin and leptin receptor gene expression is not required for the onset of sexual maturation in birds. It also seems that the role for leptin in sexual development, like in rats [25], is permissive, but not decisive, in birds.

## Conclusions

We report herein the first time that an earlier sexual maturation in hens induced by long-light regime is not accompanied with an increase in serum leptin or leptin receptor gene expression in hypothalamus, or genes expression in HPG axis.

## Competing interests

The authors declare that they have no competing interests.

## Authors’ contributions

YN carried out the experiment, participated in the data collection, data analysis and interpretation, and drafted the manuscript. JL performed the analysis of the hypothalamic genes expression and hormones detection. SW helped in editing the manuscript. RZ contributed in conception, experimental design and finished the manuscript. All authors approved the final version of the manuscript for publication.
